# The stimulus control of local enclosures and barriers over head direction and place cell spatial firing

**DOI:** 10.1002/brb3.2070

**Published:** 2021-02-19

**Authors:** Anna E. Smith, Emma R. Wood, Paul A. Dudchenko

**Affiliations:** ^1^ Centre for Discovery Brain Sciences University of Edinburgh Edinburgh UK; ^2^ Division of Psychology University of Stirling Stirling UK; ^3^ University of St. Andrews St. Andrews UK

**Keywords:** head direction cells, hippocampus, navigation, place cells, spatial cognition

## Abstract

**Objective:**

Head direction cell and place cell spatially tuned firing is often anchored to salient visual landmarks on the periphery of a recording environment. What is less well understood is whether structural features of an environment, such as orientation of a maze sub‐compartment or a polarizing barrier, can likewise control spatial firing.

**Method:**

We recorded from 54 head direction cells in the medial entorhinal cortex and subicular region of male Lister Hooded rats while they explored an apparatus with four parallel or four radially arranged compartments (Experiment 1). In Experiment 2, we recorded from 130 place cells (in Lister‐ and Long‐Evans Hooded rats) and 30 head direction cells with 90° rotations of a cue card and a barrier in a single environment (Experiment 2).

**Results:**

We found that head direction cells maintained a similar preferred firing direction across four separate maze compartments even when these faced different directions (Experiment 1). However, in an environment with a single compartment, we observed that both a barrier and a cue card exerted comparable amounts of stimulus control over head direction cells and place cells (Experiment 2).

**Conclusion:**

The maintenance of a stable directional orientation across maze compartments suggests that the head direction cell system has the capacity to provide a global directional reference that allows the animal to distinguish otherwise similar maze compartments based on the compartment's orientation. A barrier is, however, capable of controlling spatially tuned firing in an environment in which it is the sole polarizing feature.

## Graphical abstract

Head direction cells maintain stable preferred firing directions across different compartments within a maze. In this way, they provide a directional reference frame that may allow disambiguation of maze compartments which face different directions.

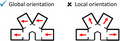

## 1 INTRODUCTION

A fundamental property of place cells, head direction (HD) cells, and grid cells is that the location or direction in which they fire is anchored to salient visual landmarks within the environment. Head direction cells are thought to provide an internal compass, while place and grid cells provide a representation of instantaneous location, and together these neural representations are thought to underpin recognition of locations and navigation (Butler et al., 2017; Thompson et al., 2018; Weiss & Derdikman, 2018). The traditional way in which a link between the spatial tuning of these cells and the external environment has been demonstrated is via the cue‐rotation manipulation. In this, spatially tuned cells are recorded in a cylindrical environment with a polarizing cue—such as a cue card—affixed to a portion of the environment wall (Muller & Kubie, 1987). The animal is then removed from the environment and the cue card is shifted radially by a fixed amount (e.g., 90°). The animal is returned to the environment and a second recording session is conducted. The same process can be repeated for a third session in which the cue card is returned to its original position. In these sessions the spatial tuning of these neurons typically follows the rotation of the landmark and its return. Thus, place fields will shift by 90° with the landmark, and then shift back when the cue card is returned to its initial position. In this way, the landmark exerts stimulus control over spatial firing.

A relatively unexplored question, however, is whether this stimulus control applies to other forms of polarizing information within the environment. Previous studies have shown that distally placed objects or landmarks are sufficient to control both spatial firing and spatial behavior (Cressant et al., 1997; Cressant et al., 1999; Dudchenko & Taube, 1997; Hamilton et al., 2008; Jayakumar et al., 2019; Knierim, 2002; Knierim et al., 1998; Lee et al., 2004; Suzuki et al., 1980), but it is unclear whether structural features of the environment are equally compelling as disambiguating references. Two studies that have looked at this have shown disparate findings: Knight et al. (2011) found that the shape of the recording enclosure did not exert consistent stimulus control over the directional firing of HD cells under normal conditions, while Clark et al. (2012) showed that the geometry of the recording environment could anchor HD cells in disoriented rats.

A related question is how head direction cells behave in environments with identical compartments. Previous studies have shown that both place cells and grid cells show repetition of their firing fields in mazes with multiple, parallel compartments (Grieves et al., 2016; Spiers et al., 2015), or with repeated alleyways (Derdikman et al., 2009). This suggests that both place cells and grid cells are driven by the local boundaries of the environment and not a global location sense. In support of this, Grieves et al. found that at a behavioral level rats have difficulty discriminating identical, connected enclosures when these are arranged in parallel (i.e., like offices in a straight hallway). A likely possibility is that place cell fields are driven by the boundaries of local environments, and thus across identical environments repetition of firing fields is expected (Barry et al., 2006; O’Keefe & Burgess, 1996).

There is evidence, however, that head direction cells are not driven by local environments. Rats readily distinguish otherwise identical maze compartments from one another when the compartments face different directions. This is seen both behaviorally and at the level of hippocampal place cell fields (Grieves et al., 2016; Harland et al., 2017). One possibility is that this capacity is underpinned by a stable “global” orientation of the head direction cell system (Dudchenko & Zinyuk, 2005; Taube & Burton, 1995; Whitlock & Derdikman, 2012; Yoder et al., 2011). Specifically, if rats possess a stable, internal directional reference across compartments of a maze, they would be able to detect the mismatch between their orientation (presumably provided by the head direction cell system) and the orientation of the compartment. The alternative view is that the head direction cell system is driven by local structural features, such as the walls of a maze compartment. If so, the preferred firing direction of a given head direction cell would shift to agree with the orientation of each compartment if the compartments face different directions.

Some hint of both of these possibilities is found in a study by Yoder et al. (2011). They showed that HD cells maintained generally stable preferred firing directions in a start box and a similarly sized goal box that were separated by 14 T‐shaped choice points. Interestingly, small but significant shifts in firing directions were observed between the start box and the second half of the maze (i.e., before the animal reached the goal box). This suggests that either the turns or the shapes of the alleyways could influence HD firing directions though not dramatically so. In an additional experiment, Yoder et al. found that HD firing directions were largely stable as rats ran from a square enclosure in one room to a novel circular enclosure in a second room. In our Experiment 1, we wished to contrast the global versus local account of HD cell orientation in a multi‐compartment environment in which the local compartments were of the same shape and equally familiar to the animal. By having compartments that were identical, but which faced different directions, we could explicitly test whether local boundaries exerted control over HD cell firing directions.

Spatially tuned neurons are sensitive to changes in the shape of the environment (Barry et al., 2007; Krupic et al., 2015; Muller & Kubie, 1987; Stensola et al., 2015; Taube et al., 1990), though this may depend on experience (Lever et al., 2002) and may reflect remapping to different contexts (Kubie & Ranck, 1983). However, the firing fields of place and grid cells close to a given wall appear more tied to it than fields farther away (Hardcastle et al., 2015; Krupic et al., 2018; see also Shapiro et al., 1997). Further, a prominent view on place cells is that their location‐specific firing arises from boundary vector cells—cells which fire at a specific distance and direction relative to a border within an environment (Barry et al., 2006; Lever et al., 2009; Solstad et al., 2008). However, it is possible that a barrier within an environment could be essential for the formation of a place field but not serve as a larger‐scale, disambiguating cue. Evidence for this is suggested by the repetition of firing fields observed in multi‐compartment environments (Derdikman et al., 2009; Grieves et al., 2016; Spiers et al., 2015) and with the introduction of repeated boundaries (Stewart et al., 2014). In these instances, the boundaries of the local environments appear to set the fields in that a field is a fixed distance and direction along the boundary, but the repetition of fields with similar, repeated boundaries (within the same contiguous space) makes identification of unique locations difficult (Grieves et al., 2016).

Finally, from the perspective of place cells, a previous study which speaks indirectly to this issue of barriers versus landmarks is that of Rivard et al. (2004). They recorded from place cells in a standard, cylindrical environment with a cue card and with a transparent plexiglass barrier. Rivard and colleagues found that place fields near the barrier followed rotations of the barrier, while those farther away from the barrier did not. A subset of the former cells also fired in a similar position relative to the barrier when it was placed in a different environment. The authors suggested that the hippocampus must thus contain two types of cells: barrier/object cells, with firing fields tied to a barrier, and traditional place cells, with place fields that encode location independent of the barrier's position. Though the barrier, and not the cue card, was manipulated in this experiment, these findings suggest that barriers and cue cards are not treated in the same way by all place cells.

To clarify how different features of the environment are encoded by spatially tuned neurons, recordings were conducted in an environment in which local rooms of a multi‐compartment apparatus could face either the same or different directions (Experiment 1). To further test whether features of the environment could exert stimulus control over head direction cells, recordings were conducted in a single environment where either a barrier or a cue card served as a sole polarizing landmark (Experiment 2). We hypothesized that if the head direction cell system allows the animal to distinguish parts of an environment that are visually and geometrically similar but differ in their orientation, then HD cells should show a constant firing direction across maze compartments. Further, if barriers and cue cards serve equally as a polarizing landmark within an environment, the stimulus control exerted by each over place cells and HD cells in a single compartment environment should be comparable. Our results suggest that, in an environment with multiple, familiar compartments, the preferred firing directions of HD cells are stable regardless of local environment. However, in an environment with a single compartment, both barriers and cue cards exert control over place‐ and head direction cell firing, although the former may be modulated by place field location.

## 2 METHOD

### 2.1 Experiment 1: Head direction cells in a maze with four rooms

#### 2.1.1 Animals

Adult, male Lister Hooded rats (*n* = 5, Charles River Laboratories, UK) weighing 320–400 g at the start of the experiment were used for the head direction cell recordings. Head direction cells were recorded in the medial entorhinal cortex (MEC, *n* = 3 animals) or the subicular complex (*n* = 2 animals). These regions were chosen as we also wished to record from boundary vector cells (which have been described in these regions), but these were not observed consistently. These animals also participated in Experiment 2.

All procedures were conducted according to the UK Animals (Scientific Procedures) Act (1986) and European Communities Council Directive of November 24, 1986 (86/609/EEC). All animal experiments were conducted in compliance with protocols approved by the University of Edinburgh Animal Welfare and Ethical Review Board (AWERB) under a UK Home Office Project License.

#### 2.1.2 Tetrodes

Tetrodes were constructed from four HML‐coated, heat annealed 17μm platinum‐iridium wires (Axona, St Albans, UK). For the place cell recordings, tetrodes were attached to drives built with Mill‐Max connectors (Mill‐Max, Oyster Bay, NY; eight tetrodes per animal) and implanted unilaterally. For the head direction cell recordings, tetrodes were attached to prefabricated Axona microdrives (Axona, St Albans, UK; four tetrodes per rat). Tetrode bundles, once assembled on microdrives, were gold‐plated (Gold Plating Solution, NeuraLynx, Ireland) to reduce the impedance to or near a target of 200 kΩ.

#### 2.1.3 Surgical procedures

Rats were anesthetized using inhalation of isoflurane (Vetflurane, Virbac, UK) delivered in medical oxygen. Intraoperative analgesia (Rimadyl, Pfizer, UK) was given at the start of the surgery. Electrodes were implanted in either the MEC or subicular complex (Experiment 1), or the CA1 cell layer of the hippocampus (Experiment 2; see Table 1). All implants were in the left hemisphere based on coordinates derived from previous implants. Previous work has shown that the properties of HD cells do not differ between hemispheres (Giocomo et al., 2014).

**TABLE 1:** Surgical coordinates for Experiments 1 and 2

Rat	Target region	AP	ML	DV	Angle
H9023	CA1	−3.5 mm from bregma	−2.4 mm from bregma	−1.7 mm from dura	None
H9024	CA1	−3.5 mm from bregma	−2.4 mm from bregma	−1.7 mm from dura	None
H9025	CA1	−3.5 mm from bregma	−2.4 mm from bregma	−1.7 mm from dura	None
H9053	CA1	−3.5 mm from bregma	−2.4 mm from bregma	−1.65 mm from dura	None
G9304	MEC	+0.4 mm from transverse sinus	−4.0 mm from lambda	−1.5 mm from dura	10° anterior
G9309	MEC	+0.8 mm from transverse sinus	−4.5 mm from lambda	−1.5 mm from dura	10° anterior
G9313	MEC	+0.3 mm from transverse sinus	−4.5 mm from lambda	−1.8 mm from dura	8° anterior
G9317	Subicular complex	−5.9 mm from bregma	−3.0 mm from lambda	−1.8 mm from dura	None
G9320	Subicular complex	−5.9 mm from bregma	−3.0 mm from lambda	−1.8 mm from dura	None

The electrode drive was secured using skull screws embedded in the skull and dental cement (Simplex Rapide, Kemdent, UK). Hydration was maintained with a bolus injection of 2.5ml 5% glucose in 0.9% w/v saline. Rats were allowed to recover for one week between surgery and the start of food restriction and recordings.

#### 2.1.4 Apparatus

##### Screening sessions

Screening sessions took place in an octagonal arena of diameter 100 cm and height 40 cm, made of wood and painted blue. In initial screening sessions, a white or striped cue card of dimensions 84 cm × 40 cm or 42 cm × 40 cm, respectively, was attached to the wall of the arena. For later screening sessions, two junk objects (a watering can and a toy sheep) were placed just above the rim of the arena, where they would be visible to the rat.

##### Parallel and radial apparatus

**FIGURE 1:**
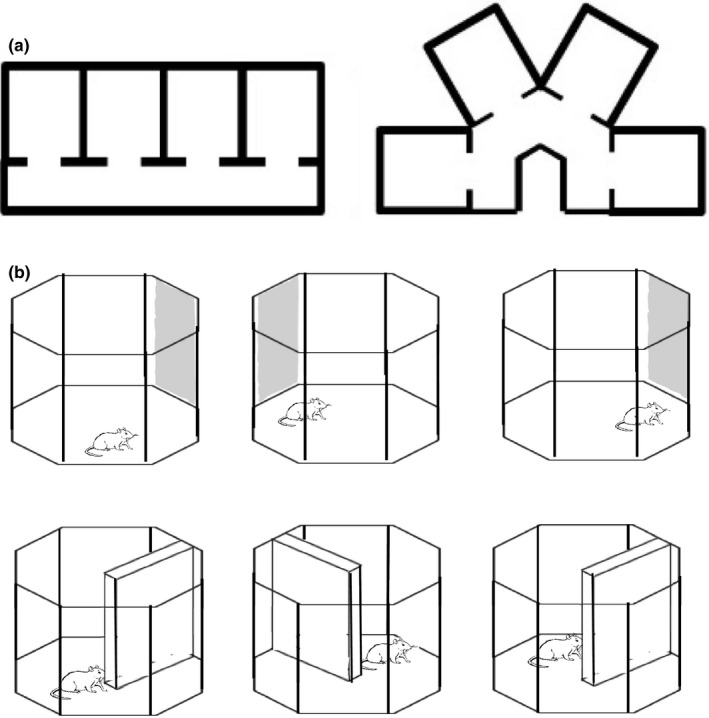
Recording environments. (a) In Experiment 1, head direction cells were recorded in an apparatus with four parallel compartments and four‐compartments arranged radially at a 60° angle to one another. (b) In Experiment 2, the stimulus control exerted by a cue card affixed to the periphery of the environment was compared with that of a barrier for the directional tuning of place and head direction cells

**FIGURE 2:**
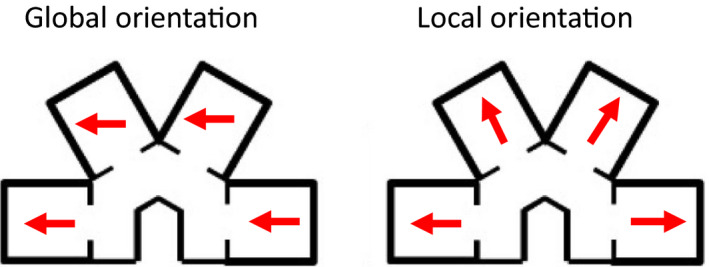
Schematic of the global versus the local orientation predictions for Experiment 1. If head direction cells encode a global directional reference, then a given head direction cell should show the same preferred firing direction in each of the four compartments of the apparatus. In contrast, if head direction cells are driven by the orientation of local compartments, then the preferred firing direction should be anchored to each compartment and thus be shifted 60° between adjacent compartments

The parallel and radial apparatus comprised four compartments of dimensions height 30 cm length 40.5 cm width 35.5 cm, which were joined by a corridor of width 20 cm and height 30 cm. Two corridors were alternated: a straight corridor, along which compartments were parallel to one another, and an inverted U‐shaped corridor, where compartments were at a 60° angle to one another (Figure 1a). These parallel and radial environments were both constructed of wood and painted blue. We hypothesized that if head direction cells provide a global orientation reference for the animal, then individual cells should maintain a similar preferred firing direction across maze compartments (Figure 2, left). In contrast, if head direction cells are driven by the local features, they should exhibit a 60° shift between compartments (and thereby maintain the same relative orientation to the compartment; Figure 2, right).

### 2.2 Experimental procedures

#### 2.2.1 Habituation

Animals were food restricted to 90%–95% (and no less than 85%) of their normal free‐feeding weight. Animals were habituated to the octagonal enclosure and trained to forage for chocolate cereal crumbs (Coco Pops, Kelloggs, UK) until they explored the arena fully over 10‐20min.

#### 2.2.2 Screening for single unit activity

Single unit activity was recorded using a 32‐channel Axona USB system (Axona, St Albans, UK). Rats were attached to the system via a flexible recording cable which allowed free movement in the arenas. Signals were passed from the cable through a ceiling mounted slip‐ring commutator (Dragonfly R&D, USA) to a preamplifier, a system unit, and desktop computer. Two infrared (hippocampus recordings) or visible‐light (MEC and subicular complex recordings) LEDs were fixed to the base of the recording cable. These were detected by a ceiling‐mounted camera to allow tracking of the animal's position and (in the case of MEC and subicular complex recordings) head direction. During screening sessions, cellular activity was recorded during this exploration and then analyzed. If no spatially tuned cells were discovered, electrodes were lowered by 50µm and screening was repeated after a delay of at least six hours.

##### Control of distal cues

In both Experiment 1 and 2, the arena or apparatus was surrounded by a floor‐to‐ceiling length black curtain to eliminate visual distal cues. White noise was played over a loudspeaker positioned in the center of the ceiling to mask unintentional auditory cues.

Upon discovery of a spatially tuned cell, animals were carried from their home cage to the arena in a covered bucket using a random walking route that varied each time and then carried once around the perimeter of the arena before testing started. This was intended to limit any maintenance of orientation between the home cage and the recording room.

##### Parallel and radial apparatus experiments

Recording experiments were composed of four sessions: a standard session (in the screening environment), a session in the parallel environment, a session in the radial environment, and a final standard session again in the screening environment. Each lasted 20–25 min and the inter‐session interval was 5–8 min. Between sessions, animals were placed in a covered holding bucket within the black curtains while the arena was swapped over. On some experimental days animals were placed in the radial apparatus before the parallel apparatus. This was varied between animals and across days.

### 2.3 Data analysis

#### 2.3.1 Cluster cutting

Once recording was complete, spike data were analyzed offline using custom Matlab scripts. The Klustakwik sorting algorithms (Kadir et al., 2014) were used to sort spikes according to energy, first principal component, peak amplitude, time at peak, and width of waveform. Clusters of spikes were then viewed and edited using Klusters software (Hazan et al., 2006). Clusters which were not recognizable as single neurons were removed, and clusters believed to represent single units were edited to remove noise spikes. A further custom Matlab script was then used to calculate isolation distance, L‐ratio, and signal‐to‐noise ratio (Schmitzer‐Torbert et al., 2005; Skaggs et al., 1993).

#### 2.3.2 Head direction cell identification and analysis

A unit was classified as a head direction cell if it satisfied the following criteria: directional tuning defined as a mean vector (*r* value) of ≥0.2 and a mean firing rate ≥0.3 Hz in the two octagon sessions that bracketed the maze sessions. The *r* ≥ 0.2 threshold was based on values commonly reported in the literature at which cells are typically above the 95th percentile of a shuffled distribution (Diehl et al., 2017; Giocomo et al., 2014; Jacob et al., 2017). For the parallel and radial compartments, head direction cell firing was compared across adjacent compartments with rotational cross‐correlations (compartment 1 versus 2, 2 versus 3, and 3 versus 4). Briefly, the firing rate for the first compartment was shifted in increments of 1° relative to the second compartment (Levin, 2019). The shift that produced the greatest Pearson correlation value was taken to be the angle of head direction shift between the two sessions. The shift in directional arrays that produced the greatest Pearson correlation value was taken to be the angle of head direction shift between compartments.

### 2.4 Experiment 2: Barrier and landmark cue rotation

#### 2.4.1 Animals

##### Place cell recordings

In addition to the animals described in Experiment 1, recordings from the CA1 cell layer of the hippocampus were conducted in four male rats (three Lister Hooded rats, and one Long‐Evans Hooded rat). The latter was included as it had taken part in an unrelated experiment previously and still had place cells. These weighed 300–350 g at the start of the experiment.

#### 2.4.2 Apparatus

##### Cue and barrier rotations

For cue card rotation manipulation, a novel 42 x 40 cm white cue card with three horizontal black stripes of equal thickness served as the landmark. For barrier rotations, a novel barrier of dimensions height 42 cm length 70 cm width 1.2 cm was inserted into the octagonal arena so that it created two regions of equal dimensions that the rat could move between by way of the gap between the end of the barrier and the facing wall (Figure 1b). The barrier was made of wood and painted the same shade of blue as the arena.

##### Cue and barrier rotation experiments

Animals were habituated to the recording environment with the cue card and barrier prior to recording taking place, as research has shown that prior exposure is required for the head direction system to rely on a landmark as sufficiently salient to form an anchor for tuning (Goodridge et al., 1998). During recordings, animals experienced either two (place cell recordings) or three (head direction cell recordings) sessions with each cue type, for a total of four or six recordings sessions on a given recording day. The order of these sessions (i.e., whether animals saw the cue or the barrier first on a given day) was varied pseudorandomly between days. In the first session of a recording day, the rat foraged for randomly scattered chocolate cereal pellets (Coco Pops, Kelloggs, UK) in the arena with either the cue card or the barrier. The rat was then removed from the arena and placed in a holding bucket. The arena was cleaned with soapy water and dried, and the cue or barrier was rotated by 90° clockwise or anticlockwise. The rat was returned to the arena for another recording session, before being returned again to the covered bucket while the arena was cleaned. For the head direction cell experiments, the cue or barrier was rotated back to its original position and the rat was returned to the arena for a third foraging session. For the place cell recordings, this session was not included as rats tended not to forage reliably after four recording sessions (two with the cue card and two with the barrier). The experiment was repeated with whichever stimulus (cue or barrier) had not been used in the first sessions. The sessions were 20–30 min long and the typical intersession interval was 4 min.

##### Identification of place cells

Place cells had to satisfy the following criteria during the first recording session of a rotation series: Each cell had to have a spatial information index greater than 0.5 bits/spike, a mean firing rate between 0.15 Hz and 6 Hz, and spike width greater than 0.25 ms. We applied a speed filter so that only spikes that were recorded when the rat was moving (>3 cm/s) were included in the analysis. As our interest was in how place fields are anchored to features of the environment, we wished to exclude the extrafield firing observed during pauses in movement (Johnson & Redish, 2007). This is consistent with previous work (e.g., Miao et al., 2015; Ólafsdóttir et al., 2015; Spiers et al., 2017; Wang et al., 2012) and is based on evidence that non‐local hippocampal replay occurs during periods of immobility (Karlsson & Frank, 2009).

##### Place cell rotation analysis

Firing‐rate maps for the rotation session were analyzed by comparing the baseline recording session to the 90° rotation session for the cue card and the barrier rotations, respectively. For each pair of sessions, the firing‐rate maps were overlaid and rotated relative to one another in 5° increments. At each angle of rotation, the Pearson's correlation between the two maps was calculated, resulting in values for maximum and minimum correlation and the angle of rotation at which the maximum correlation was achieved. The angle of rotation at which the maximum correlation was achieved was taken as the angle of place field rotation.

##### Identification of head direction cells

HD cells were identified as in Experiment 1 and were also required to have at least 100 spikes in every session.

##### Head direction cell cross‐correlation

The amount of shift in a cell's preferred firing direction (PFD) between barrier or cue sessions was calculated using a cross‐correlation method (Levin, 2019). Cross‐correlations were conducted for the following comparisons in both barrier and cue card sessions: standard session versus 90° rotation session, 90° rotation session versus return‐to‐standard session, and standard session versus return‐to‐standard session.

#### 2.4.3 Histology

At the end of the experiment, animals were terminally anesthetized with sodium pentobarbital and then perfused with phosphate‐buffered saline followed by 4% formalin. The position of the electrode was marked by passing a 25 mA current for 2 s through one tetrode. Brains were extracted and incubated in 4% formalin for at least 48 hr before being immersed in 30% sucrose (Sigma, UK) in PBS for 72 hr at 4°C. Brains were then frozen and then cut into 40 µm coronal (for hippocampal and subicular complex electrode implants) or sagittal (for MEC electrode implants) sections at the level of the region of interest. The sections were stained with Nissl stain (0.1% cresyl violet solution, Sigma, UK) and coverslipped. Sections were examined using a microscope (Leica BMRB, Germany), a QICAM camera (QImaging, Canada), and ImagePro software (Media Cybernetics, USA). Images were taken and used to confirm placement of the electrode within the target region.

#### 2.4.4 Quantification and statistical analysis

Circular statistics were performed using Oriana version 4 (Kovach Computing Services, Anglesey, UK). Values for head direction shifts or place cell rotations between sessions were plotted on a polar histogram. A Watson's *U* ^2^ test was used to determine whether the sample fitted the von Mises distribution (the circular equivalent of the normal distribution). In most cases, data were found to deviate from the von Mises distribution, and so nonparametric tests were performed. A Kuiper's test was used to determine whether the values for shifts were uniformly distributed around the circle. *V*‐tests were used to determine whether the data were significantly clustered around a predicted value. Moore's paired tests were used to determine whether head direction and place field shifts differed significantly between cue and barrier sessions. Finally, the amount of dispersion in the circular data was quantified using the concentration parameter, *κ*. Values for *κ* were compared between groups (Mardia & Jupp, 2000) to test for significant differences in variability. One qualification for this last analysis is that our data did not always fit a von Mises distribution (as the test assumes), though the results were consistent with those from the tests above.

## 3 RESULTS

### 3.1 Experiment 1

#### 3.1.1 Head direction cells maintain a stable preferred firing direction across maze compartments

**FIGURE 3:**
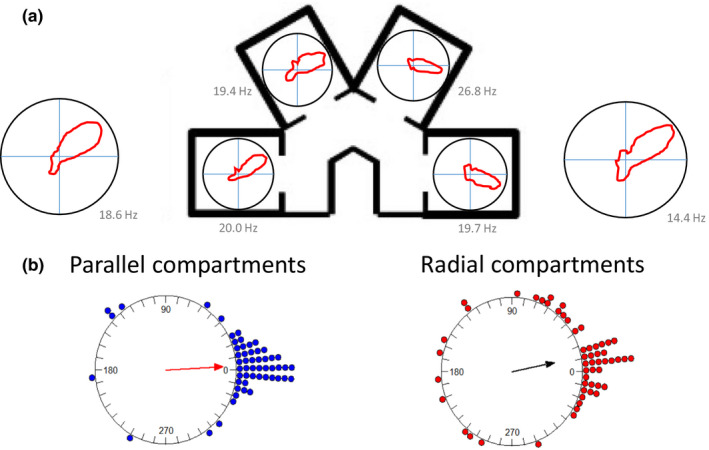
Example head direction cells response and distribution of responses in the four‐compartment environments. (a) In this example, the head direction cell exhibits a relatively stable firing direction across maze compartments, and similar preferred firing direction both before and after the maze recording session. (b) Mean distribution of head direction cell shifts between compartments for the parallel configuration (blue dots) and the radial configuration (red dots) of the environment. Solid arrows show the length and direction of the mean vector. The circular axis is the shift in cell preferred firing direction. Overall, there was not strong evidence for substantial shifts between maze compartments, and the small shifts that did occur in the radially arranged compartments were also observed in the parallel compartments. This suggests that the head direction cells maintained a global, as opposed to a local, directional reference

Figure 3a presents a head direction cell with a stable firing direction in the octagon recording sessions both before and after the multi‐compartment maze (far left and far right polar plots). Within the maze, directional firing was assessed in each compartment individually. As is evident in the figure, a stable preferred firing direction was observed in compartments 1 and 2, and a small shift was observed in compartments 3 and 4. Importantly, if the HD cell directional firing was anchored to the local compartments, large shifts of 60° would have been expected between adjacent compartments.

To quantify these observations, the mean shift in preferred firing direction was calculated for each cell for the shifts between adjacent compartments (compartment 1 versus 2, 2 versus 3, and 3 versus 4; *n* = 54 cells). Across animals, we observed that for both the parallel and the radial environments the shifts in preferred firing directions between adjacent compartments were concentrated at 0° (Figure 3b; *V*‐test comparison to 0°: parallel: *u* = 8.32, *p* < .005; radial: *u* = 6.20, *p* < .005). Thus, for both environments, the preferred firing directions of HD cells were largely stable. Somewhat more variability was observed across the radial compartments compared with the parallel compartments, however. Statistically, this was supported by a significant difference in how cells shift in the two maze configurations (Moore's test: R’ = 1.21, *p* < .025). Nonetheless, comparison of the concentration parameters (a circular measure of variability) between the radial (*κ* = 1.55) and the parallel (*κ* = 2.89) compartments did not reach significance (*F*_53.53_ = 0.23, *p* > .05).

### 3.2 Experiment 2

#### 3.2.1 Head direction cells

**FIGURE 4:**
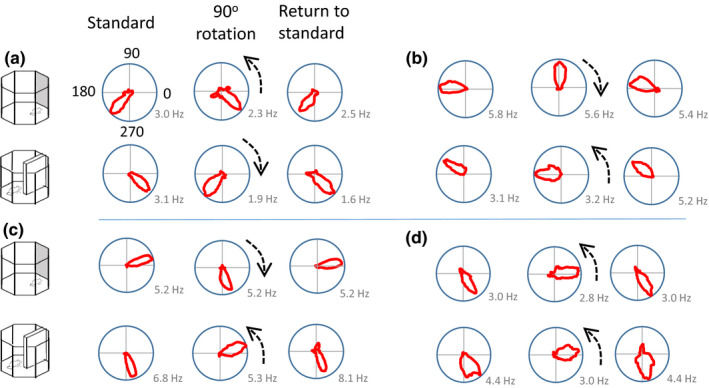
Examples of head direction tuning curve rotations in the cue card and barrier sessions. (a) An example of a cell that rotated with the anticlockwise 90° rotation of the cue card, and the 90° clockwise rotation of the barrier. (b) In this example, the cell rotated with the cue card, and showed only a small rotation with the barrier. (c) An example of a cell that rotated with both the cue card and the barrier. (d) An example of a cell that rotated by comparable amounts with both the cue card and the barrier rotations. Dashed arrows show the normalized direction of cue or barrier rotation

Thirty head direction cells were identified in 5 rats. Upon identification of a candidate head direction cell, six recording trials were conducted (three for the cue card rotation and three for the barrier rotation). In most instances, rotation of the cue card by 90° was associated with a corresponding shift in the preferred firing direction of the head direction cells (Figure 4a, c and d). On occasion, under‐rotation was observed with the barrier, while full rotation was apparent with the cue card (Figure 4b).

#### 3.2.2 Overall, comparable rotations were observed following the cue card and the barrier shifts

**FIGURE 5:**
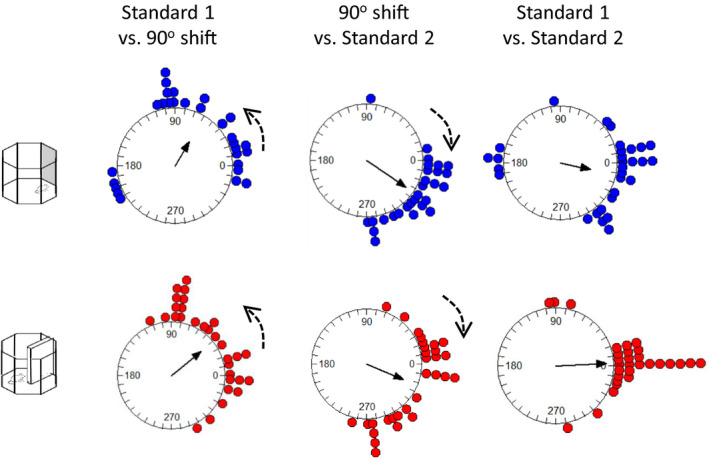
Distribution of shifts in HD cell preferred firing direction for the cue card and barrier rotation. Left plots: absolute shifts in firing direction for standard to 90° rotation session. Middle plots: absolute shifts in firing direction between the 90° rotation session and the return‐to‐standard session. Right plots: absolute shifts in firing direction between the first standard session and the return‐to‐standard session. Dashed arrows show direction of cue or barrier rotation

The examples described above suggest that slightly different patterns of stimulus control could have been observed in different recording sessions. To test whether these patterns were systematic, we pooled the data for all animals and all head direction cells. We then compared the shifts in individual head direction cells in three comparisons: the initial standard session versus a 90° rotation, 90° rotation versus postrotation standard session, and initial standard session versus postrotation standard session. As can be seen in Figure 5, the general pattern of preferred firing direction shifts with the 90° rotation of both the cue card and the barrier was comparable and in the correct direction. The mean angle of sample (solid arrow) indicates an under‐rotation of the head direction cells. A similar pattern of results was observed for the return of the cue card to its original position: shifts in firing were in the correct direction, variable, but comparable between the cue card and the barrier. Statistically, no differences were seen in the HD firing direction shifts for the initial 90° rotation of the cue card compared with the barrier (Moore's *R*’ = 0.69, *p* > .1) or the return rotations (*R*’ = 0.76, *p* > .1). Surprisingly, a significant difference between the cue card and the barrier was observed during the initial standard versus post‐rotation standard (*R*’ = 1.11, *p* < .05). Inspection of the bottom plots in Figure 5 suggests that this may be due to more variability in HD firing direction in the cue card standard sessions (mean vector *r* = 0.53) than in the barrier sessions (mean vector *r* = 0.86). To test whether variability difference significantly between the cue and the barrier session, we analyzed the concentration parameters. On this measure, the cue (*κ* = 1.09) and barrier (*κ* = 2.03) shifts did not differ in the standard to 90° rotation (*F*(29.27) = 1.05, *p* > .05). Likewise, in the 90° rotation to return comparison, the concentration parameters did not differ significantly between cue (*κ* = 3.32) and barrier (*κ* = 2.02) recordings (*F*(29.27) = −0.59, *p* > .05). Finally, in the standard to return comparison, the concentration parameters did not differ significantly between cue (*κ* = 1.25) and barrier (*κ* = 3.79) recordings (*F*(29.29) = 1.57, *p* > .05). Overall then, the variability in preferred firing direction shift was comparable between cue and barrier sessions for each rotation. Finally, both the cue card and barrier standard‐standard comparisons were centered on 0° (*V*‐test; cue card *u* = 4.01, *p* < .005; barrier *u* = 6.63, *p* < .005). This suggests comparable stability of the HD cells in the presence of the cue card and the barrier.

#### 3.2.3 Place cells

**FIGURE 6:**
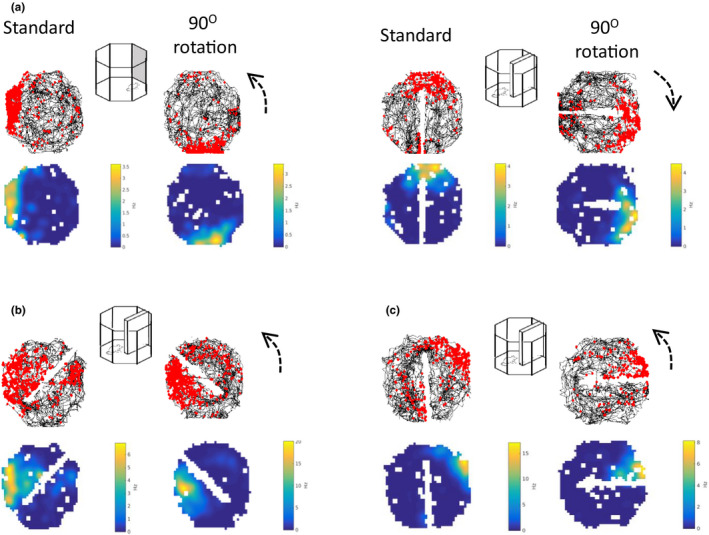
Place cell rotation examples. (a) Examples of a place field that rotates with a 90° anticlockwise rotation of the cue card and a 90° clockwise rotation of the barrier. In all examples, the top plots show the spikes (red dots) and the rat's path (black lines) during the recording session, and the bottom plots show the smoothed firing‐rate map with warmer colors indicating higher rates of firing. (b) and (c) Examples of place fields that failed to rotate with a 90° anticlockwise rotation of the barrier. Dashed arrows show direction of cue or barrier rotation

**FIGURE 7:**
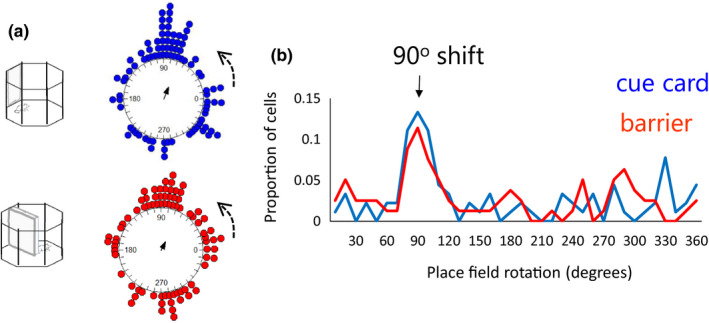
Distribution of place field shifts for the cue card and barrier rotations. (a) A similar distribution of shifts was observed for the cue card rotations (blue dots) and the barrier rotations (red dots). Solid arrows show the length and direction of the mean vector, and the axis is the amount of radial shift in the place field. (b) Proportion of shifts observed across each direction (in 10° bins). The modal shift was at 90° for both the cue card and the barrier rotations

130 place cells were identified in four animals. Following the identification of a place cell, 90° rotation sessions were conducted with both the cue card and the barrier. Example cells are shown in Figure 6. In the examples in Figure 6a, the place fields rotated with both the cue and the barrier rotations. In Figure 6b and c, examples of rotation failures with the barrier rotations are presented. The distribution of shifts is shown in Figure 7a. As is evident in the top plot, somewhat more consistent rotations were observed with the cue card (mean vector *r* = 0.31) compared with the barrier (*r* = 0.21). However, this effect did not reach statistical significance (Cue card versus barrier rotation: Moore's *R*’ = 0.354, *p* > .5). To test for differences in variability of rotations, concentration parameters (κ) were compared between the cue and barrier data. There was no significant difference in concentration between the cue (*κ* = 0.66) and barrier (*κ* = 0.44) rotations (*F*_78.89_ = 0.18, *p* > .05). As with the head direction cells, for both the cue card and the barrier rotations the distribution of place cell shifts was significantly clustered at 90° (*V*‐test versus 90°; cue card: *u* = 3.88, *p* < .005; barrier: *u* = 2.41, *p* = .008). This preponderance of 90° shifts in place fields for both the cue card and the barrier is also apparent when the proportions of cells exhibiting each shift are plotted (Figure 7b).

An additional variable that may have modulated the stimulus control of the barrier was its proximity to the place field. This led us to conduct an additional, descriptive analysis of how these responses may have varied as a function of the place field location given the previous observations of Rivard et al. (2004). First, place fields were classified as either being adjacent to the barrier (e.g., Figure 6b) or distant to the barrier (e.g., Figure 6c (pre‐rotation, left)). Then, the percentage of the fields that rotated with the barrier (within ±30° of barrier rotation) and the percentage of fields that were unchanged (<30 degree shift between baseline and barrier rotation sessions in either direction) was calculated. Only 29.4% of the fields that were distant to the barrier rotated with it, while 20.6% of the distant place fields were unchanged following this manipulation. In contrast, 48.5% of the place fields adjacent to the barrier rotated with it, while only 6.1% were unchanged following barrier rotation.

#### 3.2.4 Histology

**FIGURE 8:**
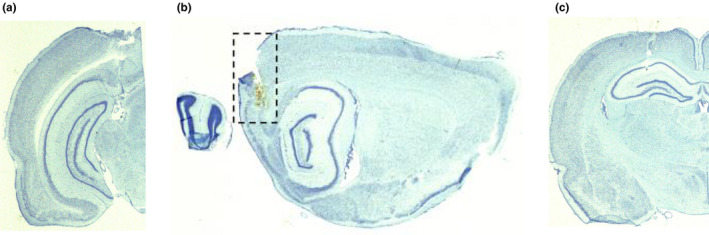
Photomicrographs of electrode placement. (a) Example of an electrode track on the subiculum/postsubiculum border. (b) Example of an electrode track in the medial entorhinal cortex. (c) Example of an electrode track in the CA1 cell layer of the hippocampus

Representative electrode tracks are presented in Figure 8. For the head direction cell recordings, electrode placements were on the subicular/postsubiculular border (Figure 8a) and in the MEC (Figure 8b). Place cell recordings were obtained from electrodes in the dorsal CA1 cell layer of the hippocampus (Figure 8c).

## 4 DISCUSSION

The current experiments were designed to test whether the preferred firing directions of head direction cells and the place fields of place cells were controlled by a polarizing structural feature of an environment. In Experiment 1, we found that a stable preferred firing direction was evident across maze compartments, both when these were parallel to one another and when adjacent compartments were offset by 60°. Despite this, a small but significant increase in firing direction variability between compartments was observed when these were arranged radially. This finding shows that in an environment in which all compartments are familiar, the local environment does not exert stimulus control over head direction cell firing directions. In Experiment 2, we observed that both a traditional cue card and a barrier exerted comparable control over HD and place cell spatial firing, though the latter was somewhat more variable and for place cells modulated by the proximity of the field to the barrier. We consider each of these findings below.

### 4.1 Head direction cells are stable across maze rooms which face different directions

In Experiment 1, we assessed the stimulus control exerted by structural features of the environment—the orientation of a local maze compartments—on head direction cells. This apparatus could be configured with either parallel compartments or radially arranged compartments. Previous recordings with place cells strongly imply that place fields are driven by both the boundaries of the local compartments and their orientation (Grieves et al., 2016; Spiers et al., 2015). In the latter experiment, place cells showed repetition of fields across identical maze compartments when they were arranged in parallel, but not when the same compartments were arranged radially (as in Experiment 1). Further, animals had difficulty discriminating the parallel compartments from one another, as one may expect if place cells fire in the same way in each compartment, but had little difficulty when the compartments were arranged radially. Indirect evidence suggests that the head direction cell system underlies this capacity. Lesions to the lateral mammillary nuclei, a key node in the head direction cell network, result in increased place field repetition in compartments facing different directions (Harland et al., 2017) and impair the ability to tell these radially arranged compartments apart (Smith et al., 2019).

The current results provide an important confirmation of this view. To allow the place cell system to disambiguate maze compartments facing different directions, a directional reference must be stable across compartments. This would allow the animal to perceive the difference in the orientation of each compartment. Our results suggest that the head direction cell system is stable across maze compartments facing different directions, and thus is capable of providing a directional reference to underlie location representation and spatial behavior (see also Whitlock & Derdikman, 2012). Implicit in this suggestion is that the behavior of individual head direction cells reflects that of the entire head direction cell system. However, even if there are multiple representations of direction in the brain, it can be assumed that a representation that provides a stable directional reference would be of most utility during navigation (Dudchenko et al., 2019).

### 4.2 A polarizing landmark and a barrier appear to exert comparable stimulus control over head direction cells

In Experiment 2, the overall pattern of results suggests that a barrier that transects the center of a single environment exerts similar control over the preferred firing direction of HD cells to that of a traditional cue card. Thus, although head direction cells did not show local anchoring to compartments in Experiment 1, we did observe that a structural feature such as a barrier was sufficient to control directional firing if this feature served as the sole polarizing landmark within an environment. At the level of individual cells, however, we observed examples of under‐rotation with the barrier in cells which rotated with the cue card (e.g., Figure 4b). Given the presumed salience of the barrier, such failures were surprising. However, a similar lack of cue control was previously observed by Knight et al. (2011). They found that, in non‐disoriented rats, HD cell firing directions failed to shift when a recording environment with a polarizing geometry (e.g., a triangle or a trapezoid) was rotated. This result contrasts with that of Clark et al. (2012), who showed that a trapezoid‐shaped environment exerted control over HD cells, but only in disoriented rats. Our results show hints of both findings. We did not explicitly disorient our animals between rotation sessions, but occasional lapses in stimulus control were observed with the barrier. Though overall the pattern of rotations was similar between the landmark and the barrier, the results from additional recording in place cells suggest that this control can be variable.

### 4.3 A barrier's stimulus control over place fields appeared comparable to that of a cue card

For the place cell recordings, rotations of the cue card and of the barrier were comparable, though variability was observed (Figure 5). Two aspects of these findings are of note. First, even for the cue card rotations, stimulus control was variable. While a number of place fields rotated a corresponding amount with the cue card, many fields failed to rotate, or shifted by a non‐corresponding amount. This lack of consistent control differs from that traditionally observed with distally placed landmarks such as a cue card. For example, Muller and Kubie (1987) found that over 15 cue card rotation sessions the average difference between the amount of rotation in the place field and the rotation of the cue card was only 3.8°. One possibility, however, may lie in the difference in the recording environments. While in the Muller and Kubie protocol recordings were done in a cylindrical enclosure, our recordings were done in an octagonal enclosure. Though unintended, it is possible that the corners of the octagon in some instances served as a cue to anchor place fields.^1^ Indeed, a number of the erroneous rotations we observed were in angles of 45° or 90°. Such an account could yield weaker control by the manipulated stimulus compared to that observed in a cylinder.

A second, qualitative observation is that place field rotations with rotations of the barrier appeared more consistent for fields close to the barrier, as opposed to those farther away. This may contribute to the variability in place field shifts observed with rotations of the barrier (Figure 7a). As described in the Introduction, previous work by Rivard et al. (2004) has shown that place fields close to a clear plexiglass barrier placed within a cylinder tended to shift with a 45° rotation of the barrier or with its translation to the other side of the cylinder. Indeed, some place fields stayed tied to the barrier even when it was placed in a second environment. Rivard et al. thus proposed that within the hippocampus there are traditional, allocentric place cells, and a second class of object/barrier cells.

Such an account, however, raises the question of how place cell responses compare to those of head direction cells. One might anticipate that the stimulus control over spatial firing would be unitary: if an animal perceives a landmark and if it is stable, the landmark should serve as an anchor for the allocentric representations of location and direction in the brain. The more variable responses of place fields in the current manipulations suggest that these may be modulated by their proximity to the barrier (see also Fenton et al., 2000), whereas HD cells respond to barriers and a cue card in a comparable way. In support of this view, there is evidence that HD cells may be more tied to distal landmarks than place cells (Yoganarasimha et al., 2006).

## 5 SUMMARY

The current experiments make two contributions. First, they show that head direction cells maintain a stable directional orientation across local enclosures that face different directions. This evidence adds weight to the view that the head direction cell system provides a key directional reference that underpins the place cell system's capacity to encode identical compartments uniquely. Such a representation, in turn, likely enables the animal to distinguish otherwise similar local environments based on the compartment's orientation. This complements the findings of Yoder et al. (2011) and extends them by showing that head direction cells maintain a stable firing direction 1) across equally familiar maze compartments, and 2) across maze compartments that are identical but oriented in a different direction.

Second, the current results demonstrate that a polarizing border within an environment *is* capable of exerting stimulus control over the spatial firing of head direction and place cells. This anchoring is comparable to that shown by a traditional “distal” landmark—a cue card fixed to a portion of the recording environment periphery. However, this control likely requires an environment in which there are no competing polarizing landmarks, and thus the local environment, in terms of the brain's representation of space, is equivalent to the global environment.

## CONFLICT OF INTEREST

The authors have no conflicts of interest to declare.

## AUTHOR CONTRIBUTIONS

A. E. S., E. R. W., and P. A. D. contributed to the conceptualization of these experiments. A. E. S. conducted the recordings. A. E. S, E. R. W., and P. A. D. contributed to the analyses of the data and the write up of the manuscript.

### PEER REVIEW

The peer review history for this article is available at https://publons.com/publon/10.1002/brb3.2070.

## Data Availability

The data from these experiments is available at the University of Stirling DataSTORRE online repository.

## Footnotes

^1^ Such an effect was hinted at in Muller and Kubie's seminal 1987 paper. Though they observed strong stimulus control by the cue card, there was more variability in the rotation sessions than in pairs of standard sessions. They suggest that “features of the environment besides the cue card may act as partial determinants of the angular coordinate of fields” (pg. 1,966).
